# Total thyroidectomy (Tx) versus thionamides (antithyroid drugs) in patients with moderate-to-severe Graves’ ophthalmopathy – a 1-year follow-up: study protocol for a randomized controlled trial

**DOI:** 10.1186/s13063-018-2876-0

**Published:** 2018-09-15

**Authors:** Lindsay Brammen, Philipp Riss, Julius Lukas, Alois Gessl, Daniela Dunkler, Shuren Li, Asha Leisser, Sandra Rezar-Dreindl, Katharina Eibenberger, Andreas Selberherr, Christian Scheuba, Andrea Papp

**Affiliations:** 1 Section of Endocrine Surgery, Department of Surgery, Medical University Vienna, Vienna, Austria; 2 Department of Ophthalmology, Medical University Vienna, Vienna, Austria; 3 Department of Internal Medicine- Section of Endocrinology, Medical University Vienna, Vienna, Austria; 4 Section for Clinical Biometrics, CeMSIIS, Medical University Vienna, Vienna, Austria; 5 Department of Nuclear Medicine, Medical University Vienna, Vienna, Austria; 6 Medical University Vienna, General Hospital Vienna, Waehringer Guertel 18-20, A-1090 Vienna, Austria

**Keywords:** Graves’ disease, Hyperthyroidism, Graves’ ophthalmopathy, Antithyroid drugs, Thyroidectomy, CAScore, NOSPECS, Muscle index, Superonasal index

## Abstract

**Background:**

Graves’ disease (GD) is characterized by thyrotoxicosis and goiter and arises through circulating autoantibodies that bind to, and stimulate, the thyroid hormone receptor (TSHR). A temporal relation between the onset of hyperthyroidism and the onset of ophthalmopathy, a common extrathyroidal manifestation, has been demonstrated. Graves’ ophthalmopathy (GO) is typically characterized by an inflammation and expansion of the extraocular muscles and an increase in retroorbital fat. There are currently three forms of therapies offered for hyperthyroidism caused by Graves’ disease: antithyroid drugs (ATD) (thionamides), radioiodine ablation (RAI) and thyroidectomy (Tx). To date, there is no clear recommendation on the treatment of Graves’ disease and GO, mainly due to the individuality of the disease in each patient. The aim of the study is to examine the difference in the outcome of GO in patients with moderate-to-severe GO who receive Tx versus further ATD after suffering their first relapse of GO, or in which GO stays the same following the initial decrease in ATD therapy after 6 months.

**Methods/Design:**

This prospective randomized clinical trial with observer-blinded analysis will analyze 60 patients with moderate-to-severe GO who receive Tx versus ATD without surgery. Main outcome variables include: muscle index measurements via ultrasound and thyroid antibody levels. Additional outcome variables include: Clinical Activity Score (CAScore), NOSPECS score, superonasal index measurements via ultrasound, and quality of life score.

**Discussion:**

This study should allow for better therapeutic choices in patients with moderate-to-severe GO. In addition, it should demonstrate whether the outcome of GO in patients with moderate-to-severe GO is better in those who receive early Tx versus further ATD. Furthermore, this study will aim to establish a standard glucocorticoid scheme before and after Tx in patients with moderate-to-severe EO.

**Trial registration:**

Eudra-CT: 2015–003515-38; Medical University of Vienna Protocol Record 1839/2015.

Date of Ethics Committee approval: 19 January 2017.

Registered on 27 January 2017.

**Electronic supplementary material:**

The online version of this article (10.1186/s13063-018-2876-0) contains supplementary material, which is available to authorized users.

## Background

### Medical problem

Graves’ disease is characterized by thyrotoxicosis and goiter and arises through circulating autoantibodies that bind to, and stimulate, the thyroid hormone receptor (TSHR) [1]. The annual incidence lies at 14 per 100,000 [2] and it is more frequent in women, with a ratio of approximately 4–6:1 [3]. The disease can also cause extrathyroidal manifestations, the most common one being ophthalmopathy [1].

Graves’ ophthalmopathy (GO) is typically characterized by an inflammation and expansion of the extraocular muscles and an increase in retroorbital fat [4]. The interaction between orbital fibroblasts and CD4+ lymphocytes has led researchers to believe that corticosteroids can be used as a standard therapy to suppress this immune reaction [5]. Swelling and enlargement of the orbital tissues can result in an increase in intraorbital pressure causing exophthalmos and venous engorgement. The different stages of GO are classified according to the European Group on Graves’ Orbitopathy (EUGOGO) [6]: mild, moderate to severe and sight-threatening.

The Clinical Activity Score (CAScore) has been previouly  published by Mourits et al. [7]. A CAScore of ≥ 3/7 indicates active disease [6] and a score of 4 or higher has an 80% positive predictive value and 64% negative predictive value in the response of patients to corticosteroid therapy. Another classification, No signs/symptoms, Only signs/no symptoms, Soft tissue involvement, Proptosis, Extraocular muscle involvement, Corneal involvement, Sight loss (NOSPECS), was first described by Werner in 1977 [8].

Risk factors associated with the development of GO include gender (female to male ratio: 2:1), cigarette smoking (also associated with relapse after therapy with antithyroid drugs (ATD) and deterioration of GO following radioablation therapy (RAI)), severity of hyperthyroidism and the degree of autoimmunity [9].

The best treatment should offer fast relief of clinical symptoms by eradicating the cause of thyrotoxicosis with only minimal risk for the patient [10]. Patients with moderate-to-severe and active GO preferably receive intravenously administered glucocorticoids as first-line therapy. If this does not work, second-line therapy includes a second course of high-dose intravenously administered glucocorticoids, orally administered glucocorticoids in combination with cyclosporine and possibly orbital radiotherapy [6, 11]. In terms of the dosages and length of glucocorticoid therapy, there have been few studies published to date. A study by Bartalena et al. suggested a starting dose of 80–100 mg with a gradual tapering and withdrawal over 5–6 months in oral glucocorticoid therapy [12]. Regarding intravenously administered glucocorticoid therapy, two studies delivered 15 mg/kg for the first four infusions followed by 7.5 mg/kg for the remaining eight infusions for a total of 12 infusions over 10 weeks [13, 14]. However, there is currently no consensus on the dosage or therapy length of either orally or intravenously administered glucocorticoid therapy.

The three types of therapies offered for Graves’ disease are ATD, RAI and total thyroidectomy (Tx). ATD therapy is generally well-tolerated, carries a low risk of hypothyroidism, carries no need of radiation exposure and is relatively safe to use in pregnancy and breastfeeding. However, it should only be given for a limited period of time (between 18 and 24 months) due to the side-effects, such as hepatitis and agranulocytosis, as well as a high relapse rate of up to 50% following withdrawal of the therapy [2, 15–19].

Definitive thyroid tissue ablation by RAI leads to lifelong hypothyroidism in up to 80% of patients. However, the patients are exposed to radiation which can cause a worsening of GO (radiation thyroiditis) due to a massive efflux of thyroid antigens from the thyroid that then stimulate antithyroid antibody production [19–27]. An exacerbation of preexisting ophthalmopathy can be prevented with therapy using orally administered glucocorticoids after RAI for anywhere between 6 and 12 weeks [6, 28, 29].

Thyroidectomy also provides definitive treatment, without exposure to radiation. Total thyroidectomy has also been shown to induce euthyroidism faster and more permanently (97%) than in patients who received RAI (73%), as well as less frequent persistence of GO, in accordance with other studies [6, 21, 22, 26, 27, 30]. In addition, levels of thyroid-stimulating immunoglobulins also rapidly and steadily declined in patients who received total thyroidectomy compared to those who underwent RAI or who took ATD [30]. Several studies have demonstrated a significant improvement of GO in patients who underwent thyroidectomy [10, 31, 32]. However, the patients who undergo thyroidectomy do need life-long substitution of thyroid hormone and have a neck scars [11]. Surgery is also associated with complications, such as permanent or transient recurrent laryngeal nerve palsy, permanent or transient hypoparathyroidism and neck hematoma requiring re-operation [33].

At present, there is no consensus on the treatment of Graves’ disease and GO, mainly due to the fact that the pathophysiology of the disease is still not completely understood, as well as the different courses of the disease in each patient. There are few published prospective randomized trials comparing the different therapies. To our knowledge, no studies have prospectively examined the effects of Tx and ATD on GO.

### Aim of the study

The aim of this study is to assess the outcome of GO in patients with moderate-to-severe GO who receive early Tx after their first relapse of GO or in which GO stays the same following the initial decrease in their ATD therapy after 4–6 months. Furthermore, this study will aim to establish a standard glucocorticoid scheme before and after Tx. Patients will undergo follow-up examinations for 1 year after study inclusion. The data acquired from this study will allow for better therapeutic choices in patients with GO. This study is considered a phase III study.

## Methods/design

### Objective of the study

The overall objective of this prospective randomized clinical trial with observer-blinded analysis is to assess the outcome of GO in patients with moderate-to-severe GO who receive early Tx after their first relapse of GO or in which GO stays the same following the initial decrease in their ATD therapy after 4–6 months.

The main outcome variables investigated include: muscle index (MI) measurements via ultrasound before randomization and 1 year after the conducted therapy, as well as thyroid antibody levels, measured at each follow-up appointment. Additional outcome variables such as CAScore/NOSPECS score (calculated at each appointment), superonasal index (SNI) measurements via ultrasound and quality of life score (both obtained before randomization and 1 year after the conducted therapy) will be analyzed. See Tables 4 and 5.

### Trial population

Sixty patients with moderate-to-severe GO who suffer their first relapse of GO or in which GO stays the same following the initial decrease in their ATD therapy after 4–6 months will be included.

### Clinical sites

The clinical sites included in this study are the Department of General Surgery, the Department of Endocrinology and Metabolism, the Department of Ophthalmology and the Department of Nuclear Medicine, Vienna General Hospital (academic hospital), Medical University Vienna. In order to ensure adequate participant enrollment, all clinical sites will work together meticulously. In addition, the clinical trial will be presented at other departments of surgery, endocrinology and nuclear medicine within Austria and at medical conferences to aid in achieving adequate participant enrollment. All departments will be involved in enrolling the participants. The intervention allocation will be conducted at the Department of Surgery through the Randomizer for Clinical Trials (see page 12).

### Eligibility

Detailed inclusion and exclusion criteria are specified in Table 1.

**Table 1:** Subject inclusion and exclusion criteria

Inclusion criteria	Exclusion criteria
• GD and GO onset ≤ 12 months • No previous GD treatment other than ATD • First relapse after decrease of antithyroid medication within 4–6 months • GO treatment with glucocorticoids based on the Kahaly scheme • Patients under ATD with normal thyroid function or subclinical hyperthyroid function and moderate-to-severe GO • Clinically active inflammation according to CAScore (≥ 3/7) • Informed consent	• GD and GO onset ≥ 12 months • More than one relapse of GO longer than 6 months from diagnosis • Previous GD treatment by RAI or surgery • SNI greater than 7.0 • Urgent orbital decompression surgery • Loss of vision • Loss of visual field • Loss of color vision • Patients not receiving glucocorticoids for GO • Cytological findings of postsurgical histopathological results suspicious for malignancy • Pregnancy or breastfeeding • Contraindication to GC • Halt of GC therapy • Patients with diabetes mellitus • Age below 18 years • No informed consent

Legend: *ATD* antithyroid drugs, *CAScore* Clinical Activity Score, *GC* glucocorticoid, *GD* Graves' disease, *GO* Graves' ophthalmopathy, *RAI* radioiodine ablation

### Consent

Before being admitted to the clinical study, the patient will have a pre-treatment visit to give informed consent. During this visit, the patient will be screened and informed about the study procedures, risks, benefits and data management.

### Procedures for blinding and minimizing bias

Several methods are used in order to minimize random and systematic sources of bias. To minimize selection bias, our inclusion and exclusion criteria are precisely defined. Screening lists and case report files are monitored by independent monitors from the Clinical Trials Coordination Center to ensure correct patient recruitment and meticulous documentation. In order to achieve equal groups of patients free from systematic selection bias, an Internet-based computer randomization is performed after informed consent.

The Internet-based computer randomization is executed using the Randomizer for Clinical Trials (version 1.8.1, Institute for Medical Informatics, Statistics and Documentation, Medical University of Graz, Graz, Austria). The randomization is carried out to generate equal treatment groups by minimizing selection bias. Stratification reduces bias. The trial will only be performed at the Vienna General Hospital in high-volume departments minimizing bias due to learning effects. No additional surgical training has to be done as a standard thyroidectomy will be performed. No additional ophthalmology training has to be done, as the patients will be seen by physicians specialized in the field of GO.

The study will be conducted as a prospective, randomized clinical trial with observer-masked analysis. Every patient receiving follow-up will wear a scarf on their neck to mask any thyroidectomy scars, should there be one present. The ophthalmologist who conducts the follow-up measurements will not be allowed to look into the patient’s file to see which treatment they received. Thereafter, a second non-masked ophthalmologist will resume medical evaluation, treatment and further patient guidance beyond the study protocol.

### Interventions

#### Pre-treatment interventions

At this screening visit, baseline data in the form of a case report will be documented, including past medical history, current medications, blood parameters, eye examination including slit-lamp bimicroscopy, indirect fundoscopy, visual acuity determination, Hertel exophthalmometry, ocular motility, CAScore and NOSPECS score as well as ocular ultrasound and quality of life questionnaire in German (see Additional file 1). Patients will then be randomized into one of the two treatment groups. All trial data will be collected and documented in the case report form (CRF) (see Additional file 2).

Tables 2 and 3 describe the muscle index (MI) and superonasal index (SNI).

**Table 2:** Muscle index (MI)

Normal MI:	< 5.0	
Mild Graves’ orbitopathy	4.5–5.5	Grade I
Moderate Graves’ orbitopathy	5.5–6.5	Grade II
Severe Graves’ orbitopathy	> 6.5	Grade III

**Table 3:** Superonasal index (SNI)

Normal SNI	< 5.75	
Danger for optic nerve compression	> 7.0	Grade IV

#### Kahaly scheme

All patients will initially receive glucocorticoids for 12 weeks according to the Kahaly scheme [34]. Dosage: 500 mg methylprednisolone given intravenously once a week for 6 weeks followed by a reduction to 250 mg methylprednisolone intravenously once a week for 6 weeks.

All patients will also initially receive ATD. They will come every 4–8 weeks for follow-up. Patients suffering a relapse after 4–6 months will be randomized.

#### Surgical intervention: thyroidectomy

The responsible surgeon will document the surgical intervention and any particulars that may have occurred during or following surgery. A standardized surgical approach to the thyroid will be conducted in that a total thyroidectomy will take place.

On the first post-operative day, blood analyses of calcium and parathyroid hormone will be performed. One the second post-operative day the patient will undergo a postoperative ear-nose-throat examination to determine the functionality of the recurrent laryngeal nerve on both sides following the operation.

All patients will receive intravenously administered glucocorticoids (250 mg methylprednisolone) according to our therapy scheme following surgery once a week for 4 weeks.

#### Drug intervention: ATD

Patients randomized into the ATD group will continue to receive ATD in standard dosages for their hyperthyroidism and GO. They will continue to come for follow-up roughly every 8 weeks.

These patients will also undergo a 4-week intravenous therapy with 250 mg methylprednisolone once a week.

#### Post-treatment interventions

If at any point there is an aggregation of the GO, a third glucocorticoid cycle can be administered.

**Fig. 1:**
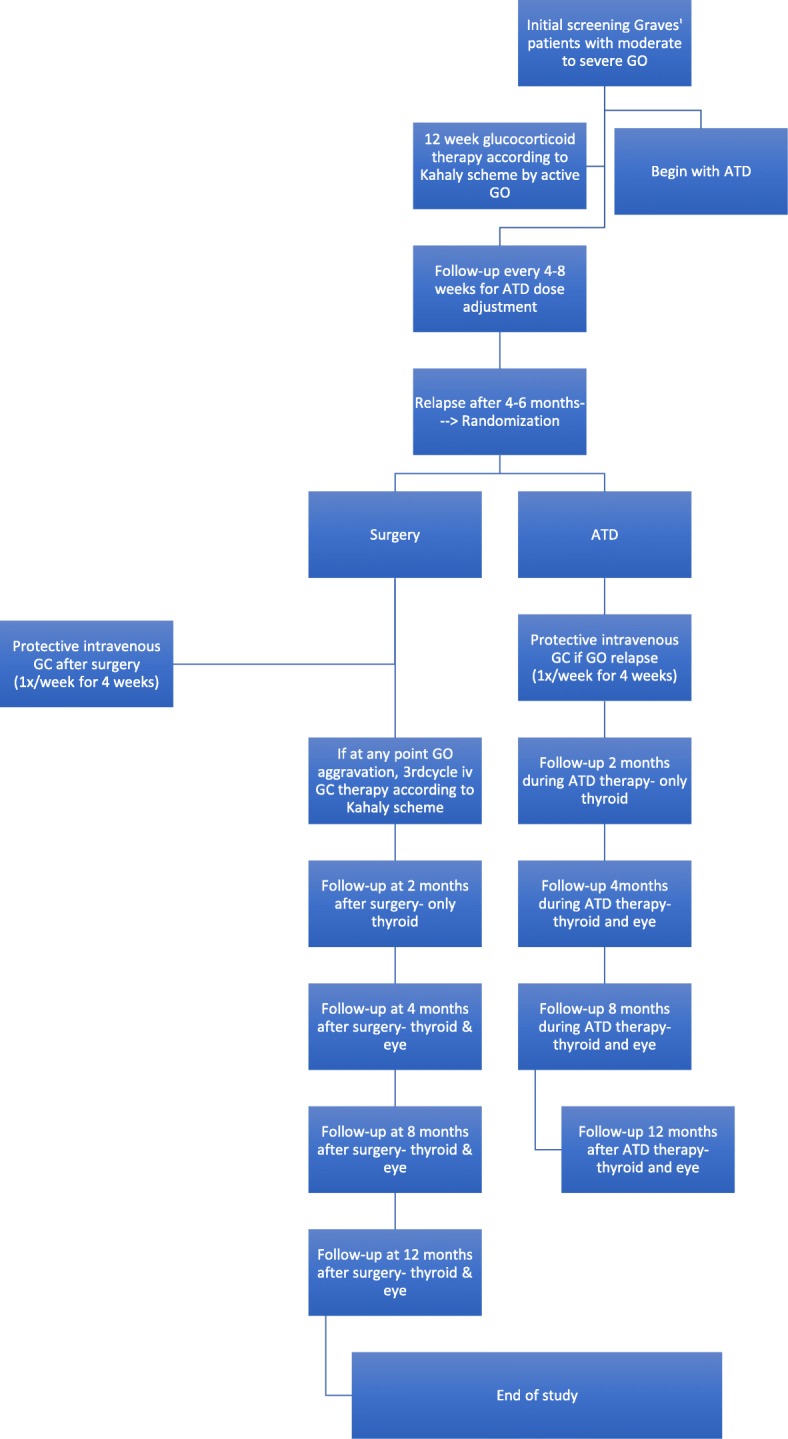
Flow-chart of the interventions

**Fig. 2:**
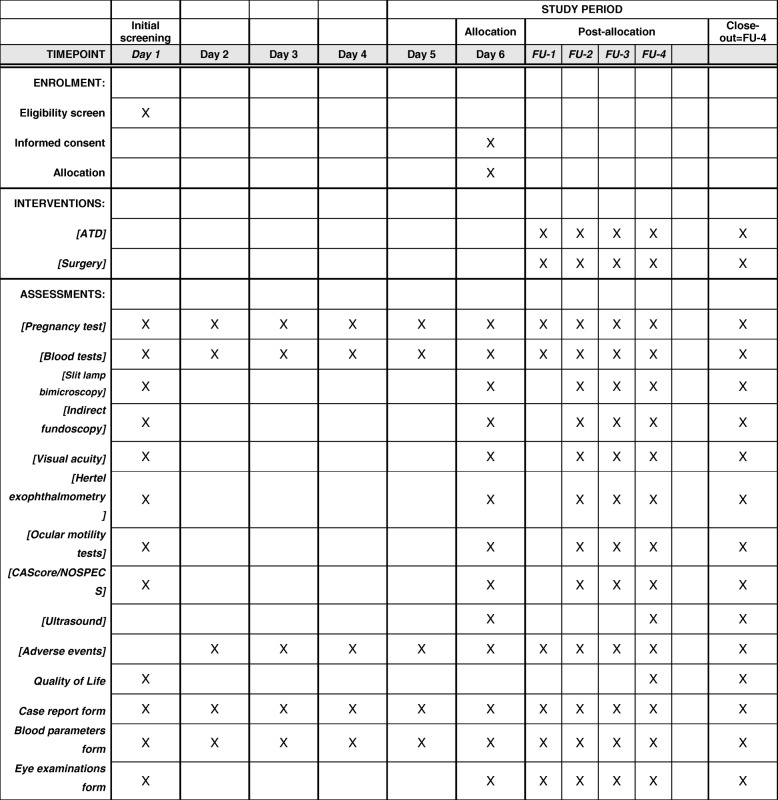
Study schedule

After both interventions, all patients will be seen in four follow-up appointments where the same parameters will be examined, as mentioned above in the pre-treatment intervention. At the first follow-up 8 weeks following treatment, a blood test will be conducted and a CRF with the blood parameters and current medications will be filled out. The second follow-up 16 weeks following treatment will include a case report with current medications, blood parameters, eye examination including slit-lamp bimicroscopy, indirect fundoscopy, visual acuity determination, Hertel exophthalmometry, ocular motility, CAScore and NOSPECS score and ocular ultrasound. The third follow-up 32 weeks following treatment contains the same case report and parameters as the second follow-up. The last follow-up 1 year after treatment will again include a case report with current medications, blood parameters, eye examination including slit-lamp bimicroscopy, indirect fundoscopy, visual acuity determination, Hertel exophthalmometry, ocular motility, CAScore and NOSPECS score and ocular ultrasound as well as the final quality of life questionnaire. Figure 1 is a flow-chart of the interventions. Figure 2 illustrates the study schedule. Tables 4 and 5 list pre-treatment and post-treatment parameters to be examined.

**Table 4:** Pre-treatment schedule and parameters examined

	Initial screening until randomization						
	Day 1 (initial screening)	Day 2 (4–8 weeks after day 1)	Day 3 (4–8 weeks after day 2)	Day 4 (4–8 weeks after ATD reduction)	Day 5 (12–16 weeks after ATD reduction)	Day 6 (20–24 weeks after ATD reduction	
Pregnancy test	X	X	X	X	X	X	Randomization and treatment
Blood tests	X	X	X	X	X	X	
Slit-lamp bimicroscopy	X					X	
Indirect fundoscopy	X					X	
Visual acuity determination with Snellen test	X					X	
Hertel exophthalmometry	X					X	
Ocular motility, cover test, prism cover test, convergence	X					X	
CAScore/NOSPECS	X					X	
Ultrasound						X	
Adverse events		X	X	X	X	X	
Quality of life form	X						
Case report form	X	X	X	X	X	X	
Blood parameter form	X	X	X	X	X	X	
Current medication form	X	X	X	X	X	X	
Eye examination form	X					X	

Legend: *ATD* antithyroid drugs, *CAScore* Clinical Activity Score, *NOSPECS* No signs/symptoms, Only signs/no symptoms, Soft tissue involvement, Proptosis, Extraocular muscle involvement, Corneal involvement, Sight loss

**Table 5:** Follow-up schedule and parameters examined

Follow-up schedule				
	Follow-up 1 (8 weeks following treatment)	Follow-up 2 (16 weeks following treatment)	Follow-up 3 (32 weeks following treatment)	Follow-up 4 (1 year following treatment)
Pregnancy test	X	X	X	X
Blood test	X	X	X	X
Slit-lamp bimicroscopy		X	X	X
Indirect fundoscopy		X	X	X
Visual acuity determination with Snellen test		X	X	X
Hertel exophthalmometry		X	X	X
Ocular motility, cover test, prism cover test, convergence		X	X	X
CAScore/NOSPECS		X	X	X
Ultrasound				X
Adverse events	X	X	X	X
Quality of life form				X
Case report form	X	X	X	X
Blood parameter form	X	X	X	X
Current medication form	X	X	X	X
Eye examination form		X	X	X

### Safety aspects

All adverse events (AE) and serious adverse events (SAE) have to be reported to the principal investigator and the local Ethics Committee, as well as the Clinical Trials Coordination Center. Patients may be withdrawn from the study at any time either at their own request or at the request of the principal investigator. All AE and SAE of both treatment groups will be demonstrated in comparison considering severity and causality in a safety analysis. Safety interim analyses are planned each year and the independent Data and Safety Board will follow the progress of the trial to control the safety. Data management will be done according to International Council for Harmonization-Good Clinical Practice (ICH-GCP). All data will be collected by the study coordinator (Dr. Brammen). All data will be pseudonymously and securely stored in order to maintain confidentiality by the study coordinator.

### Power calculation

The main study outcome variable will be muscle index (MI), where a difference of 1 corresponds to a difference of one clinical grade (grade II or grade III). We assume, that a proportion of patients (in both treatment groups) will not respond to their treatments. Therefore, an observed difference in MI of 0.7 is defined as clinically relevant in the study. A sample size of 29 in each group will have 90% power to detect a difference in mean changes of 0.7, making a conservative assumption about the standard deviation of the changes of 0.8, and using a two-group *t* test with a two-sided significance level of 0.05 (nQuery Advisor 7.0). At randomization patients will be stratified according to their MI grading (grade II versus grade III).

### Statistical analysis

The data will be analyzed according to the intention-to-treat principle. This means, that for patients with missing MI values at follow-up measurements a conservative value will be imputed. If patients have a SNI >  7 during follow-up, which requires additional treatment, later MI values will be estimated using last value carried forward. The primary and continuous secondary endpoints will be analyzed using repeated measures analysis of covariance, using the randomization group as factor and the baseline values of MI as covariate. The contrast of main interest is the difference between groups in the means computed over all post-randomization measurements. MI and SNI will be obtained before randomization and 1 year after the conducted therapy. Quality of life questionnaire will also be obtained before randomization and after 1 year of follow-up. Further binary secondary endpoints will be analyzed by Mantel-Haenszel tests stratified for the clinical MI grading at baseline. All secondary endpoints will be analyzed at the specified time points (see Tables 4 and 5) and reported and thus no correction for multiple testing is done. Any analyses that will be conducted, but which are not mentioned in the study protocol, will be interpreted as explorative. A statistical analysis plan detailing all statistical computations will be completed prior to the lock of the database. Values are given as *p* values and odds ratio (95% confidence interval); *p* values < 0.05 are considered statistically significant. Analysis of data will be performed using SPSS (Statistical Package for Social Sciences, Chicago, IL, USA) v21 for Windows.

### Trial organization, quality control and registration

Trial organization, quality control and registration were designed at the Department for General Surgery, Section Endocrine Surgery in cooperation with Clinical Biometrics, CeMSIIS, Medical University Vienna.

Quality assurance will be done in cooperation with the Network of Coordinating Centers for Clinical Trials (KKS). The trial is monitored by an independent monitor of the KKS in Vienna. The trial is performed according to the Declaration of Helsinki in its current German version and the Good Clinical Practice (GCP) recommendations. Before the start of the trial the independent Ethics Committee of the Medical University Vienna gave a positive vote (Protocol record 1839/2015). The trial was registered at the European Clinical Trials Database (Eudra-CT: 2015–003515-38).

The study will be performed in accordance with the Declaration of Helsinki (1964), including current revisions. All studies must follow the ICH-GCP Guidelines and, if applicable, The Code of Federal Regulations (USA). This study follows the EU Directive embedded in the Austrian Drug Act. The clinical trial will be performed in full compliance with the legal regulations according to the Drug Law (AMG-Arzneimittelgesetz) of the Republic of Austria. An application was also submitted to and approved by the Austrian Competent Authorities (Bundesamt für Sicherheit in Gesundheitswesen (BASG)) represented by the Agency for Health and Food Safety (AGES PharmMed).

### Current status and duration of the trial

The study protocol version 2.3 from 15 March 2018, has been approved by the local Ethics Committee. The trial will begin recruiting patients as of 1 June 2018. The recruiting period will be expected to last 24 months.

## Discussion

This clinical trial is a single-center, prospective randomized trial designed to assess which therapy method provides the better outcome in patients with moderate-to-severe GO. Since moderate-to-severe GO can be debilitating, it is essential to find a treatment method that provides the optimum therapy and best quality of life. In addition, we hope to establish a standard glucocorticoid scheme before and after Tx, given that there is, to date, no such consensus in patients with moderate-to-severe GO.

There are several advantages and disadvantages to a single-center study. Some advantages to our study center are that it is conducted in a teaching hospital with a high volume of patients and all patients are treated with the same high standard. Given that our university hospital is the largest specialized center for GO within Austria, the majority of patients are sent to us. In single-center trials, the clinical investigators are continuously involved and communication is simplified (one set team is working together on this trial with specific contact persons from each clinical unit). Also, there is reduced variability regarding trial conduct and data collection. Some disadvantages include possible recruitment issues, thus resulting in failure to demonstrate a treatment difference when one is present (type II error). In order to overcome recruitment issues, we will present the clinical trials at other hospitals and at medical conferences within Austria to secure a high recruitment rate. Given that Graves’ patients with moderate-to-severe GO should only be treated in specialized centers due to the debilitating side-effects it can cause, generalizability to other settings should not be an issue.

As has been previously mentioned and demonstrated in various studies, the experts have not been able to come to a consensus on the treatment of Graves’ disease and GO, mainly due to the individuality of the disease in each patient. In addition, the usefulness of total thyroid ablation (TTA) has yet to be confirmed by long-term randomized controlled trials (RCTs). The follow-up periods in previous studies have been too short and were not standardized. Therefore, our study will perform a follow-up in each patient at 8, 16, 32 and 52 weeks after their treatment. This will allow for an assessment of the medium- to long-term effects follow surgery or continuation of ATD.

All studies should be conducted according to the best current knowledge. A prospective, randomized clinical trial with observer-blinded analysis can be considered a “gold standard” when evaluating various medical methods and procedures. However, bias is also observed in RCTs. In surgical trials, a double-blinded trial is difficult to establish, for it would be unethical to operate on a patient with Graves’ disease and not remove their thyroid gland. As previously mentioned, all patients in follow-up will wear a scarf around their neck to hide any surgical scar, should there be one. The purpose of wearing this scarf will be emphasized to all patients at each follow-up appointment. One possible way to ensure that all patients wear a scarf is to have them meet the clinical investigator of surgery in the outpatient clinic before they go to their follow-up at the ophthalmology department.

However, patient selection, randomization, documentation, data assessment and statistical analysis are all susceptible to bias, yet are important design issue. All of these issues are specified in the clinical trial protocol (Additional file 3).

## Additional files

Additional file 1: Quality of life questionnaire in German. (DOCX 18 kb)

Additional file 2: Case report form. (DOCX 72 kb)

Additional file 3: Standard Protocol Items: Recommendations for Interventional Trials (SPIRIT) 2013 Checklist: recommended items to address in clinical trial protocol and related documents*. (PDF 1664 kb)

## Abbreviations

AE: Adverse event
AGES: Agency for Health and Food Safety
AMG: Arzneimittelgesetz (Austrian Drug Act)
ATD: Antithyroid drug
BASG: Bundesamt für Sicherheit in Gesundheitswesen (Federal Office for Safety in Health Care)
CAScore: Clinical Activity Score
CRF: Case report form
CT: Computer tomography
GC: Glucocorticoid
GD: Graves’ disease
GO: Graves’ ophthalmology
ICH-GCP: International Council for Harmonization-Good Clinical Practice
MI: Muscle index
MRI: Magnetic resonance imaging
NOSPECS: No signs/symptoms, Only signs/no symptoms, Soft tissue involvement, Proptosis, Extraocular muscle involvement, Corneal involvement, Sight loss
RAI: Radioiodine ablation
RCT: Randomized controlled trial
SAE: Severe adverse event
SNI: Superonasal index
SOP: Standard operating procedure
SUSAR: Suspected unexpected serious adverse reactions
T3: Triiodothyronine
T4: Thyroxine
TRAbs: Antibodies against TSH receptor
TSH: Thyroid-stimulating hormone
TSHR: Thyroid-stimulating hormone receptor
TTA: Total thyroid ablation
TX: Total thyroidectomy

## References

[1] Menconi F, Marcocci C, Marino M (2014). Diagnosis and classification of Graves' disease. Autoimmun Rev.

[2] Cooper GS, Stroehla BC (2003). The epidemiology of autoimmune diseases. Autoimmun Rev.

[3] Stalberg P, Svensson A, Hessman O, Akerstrom G (2008). Surgical treatment of Graves' disease: evidence-based approach. World J Surg.

[4] Burch HB, Wartofsky L (1993). Graves' ophthalmopathy: current concepts regarding pathogenesis and management. Endocr Rev.

[5] Bahn RS, Heufelder AE (1993). Pathogenesis of Graves' ophthalmopathy. N Engl J Med.

[6] Bartalena L, Baldeschi L, Dickinson A, Eckstein A (2008). Consensus statement of the European Group on Graves' orbitopathy (EUGOGO) on management of GO. Eur J Endocrinol.

[7] Mourits MP, Prummel MF, Wiersinga WM, Koornneef L (1997). Clinical activity score as a guide in the management of patients with Graves' ophthalmopathy. Clin Endocrinol.

[8] Werner SC (1977). Modification of the classification of the eye changes of Graves' disease: recommendations of the Ad Hoc Committee of the American Thyroid Association. J Clin Endocrinol Metab.

[9] Hegedus L, Bonnema SJ, Smith TJ, Brix TH (2012). Treating the thyroid in the presence of Graves' ophthalmopathy. Best Pract Res Clin Endocrinol Metab.

[10] Elsayed YA, Abdul-Latif AM, Abu-Alhuda MF, Halim HA (2009). Effect of near-total thyroidectomy on thyroid orbitopathy due to toxic goiter. World J Surg.

[11] Bartalena L (2013). Graves' orbitopathy: imperfect treatments for a rare disease. Eur Thyroid J..

[12] Bartalena L, Pinchera A, Marcocci C (2000). Management of Graves' ophthalmopathy: reality and perspectives. Endocr Rev.

[13] Menconi F, Marino M, Pinchera A, Rocchi R (2007). Effects of total thyroid ablation versus near-total thyroidectomy alone on mild to moderate Graves' orbitopathy treated with intravenous glucocorticoids. J Clin Endocrinol Metab.

[14] Moleti M, Violi MA, Montanini D, Trombetta C (2014). Radioiodine ablation of postsurgical thyroid remnants after treatment with recombinant human TSH (rhTSH) in patients with moderate-to-severe Graves' orbitopathy (GO): a prospective, randomized, single-blind clinical trial. J Clin Endocrinol Metab.

[15] Schleusener H, Schwander J, Fischer C, Holle R (1989). Prospective multicentre study on the prediction of relapse after antithyroid drug treatment in patients with Graves' disease. Acta Endocrinol.

[16] Vitti P, Rago T, Mazzeo S, Brogioni S (1995). Thyroid blood flow evaluation by color-flow Doppler sonography distinguishes Graves' disease from Hashimoto's thyroiditis. J Endocrinol Investig.

[17] Vitti P, Rago T, Chiovato L, Pallini S (1997). Clinical features of patients with Graves' disease undergoing remission after antithyroid drug treatment. Thyroid.

[18] Hegedus L (2009). Treatment of Graves' hyperthyroidism: evidence-based and emerging modalities. Endocrinol Metab Clin N Am.

[19] Gurgul E, Sowinski J (2011). Primary hyperthyroidism—diagnosis and treatment. Indications and contraindications for radioiodine therapy. Nucl Med Rev Cent East Eur.

[20] Kriss JP, Pleshakov V, Rosenblum AL, Holderness M (1967). Studies on the pathogenesis of the ophthalmopathy of Graves' disease. J Clin Endocrinol Metab.

[21] Tallstedt L, Lundell G, Torring O, Wallin G (1992). Occurrence of ophthalmopathy after treatment for Graves' hyperthyroidism. The Thyroid Study Group. N Engl J Med.

[22] Bartalena L, Marcocci C, Bogazzi F, Manetti L (1998). Relation between therapy for hyperthyroidism and the course of Graves' ophthalmopathy. N Engl J Med.

[23] Acharya SH, Avenell A, Philip S, Burr J (2008). Radioiodine therapy (RAI) for Graves' disease (GD) and the effect on ophthalmopathy: a systematic review. Clin Endocrinol.

[24] Bartalena L, Baldeschi L, Dickinson AJ, Eckstein A (2008). Consensus statement of the European group on Graves' orbitopathy (EUGOGO) on management of Graves' orbitopathy. Thyroid.

[25] Ponto KA, Zang S, Kahaly GJ (2010). The tale of radioiodine and Graves' orbitopathy. Thyroid.

[26] Li HX, Xiang N, Hu WK, Jiao XL (2016). Relation between therapy options for Graves' disease and the course of Graves' ophthalmopathy: a systematic review and meta-analysis. J Endocrinol Investig.

[27] Wu VT, Lorenzen AW, Beck AC, Reid VJ (2017). Comparative analysis of radioactive iodine versus thyroidectomy for definitive treatment of Graves’ disease. Surgery.

[28] Marcocci C, Bartalena L, Bogazzi F, Bruno-Bossio G (1992). Relationship between Graves' ophthalmopathy and type of treatment of Graves' hyperthyroidism. Thyroid.

[29] Lal G, Ituarte P, Kebebew E, Siperstein A (2005). Should total thyroidectomy become the preferred procedure for surgical management of Graves' disease?. Thyroid.

[30] Kautbally S, Alexopoulou O, Daumerie C, Jamar F (2012). Greater efficacy of total thyroidectomy versus radioiodine therapy on hyperthyroidism and thyroid-stimulating immunoglobulin levels in patients with Graves' disease previously treated with antithyroid drugs. Eur Thyroid J.

[31] Witte J, Goretzki PE, Dotzenrath C, Simon D (2000). Surgery for Graves' disease: total versus subtotal thyroidectomy-results of a prospective randomized trial. World J Surg.

[32] De Bellis A, Conzo G, Cennamo G, Pane E (2012). Time course of Graves' ophthalmopathy after total thyroidectomy alone or followed by radioiodine therapy: a 2-year longitudinal study. Endocrine.

[33] Duh QY (1999). Thyroidectomy for the treatment of Graves' disease. Thyroid.

[34] Zang S, Ponto KA, Kahaly GJ (2011). Clinical review: intravenous glucocorticoids for Graves' orbitopathy: efficacy and morbidity. J Clin Endocrinol Metab.

