# Supplementary material for: Total thyroidectomy (Tx) versus thionamides (antithyroid drugs) in patients with moderate-to-severe Graves’ ophthalmopathy – a 1-year follow-up: study protocol for a randomized controlled trial
Source: Trials. 2018 Sep 15;19:495. doi: 10.1186/s13063-018-2876-0 (PMC6139165; doi:10.1186/s13063-018-2876-0)
Supplement: Supplementary file 1 — Quality of life questionnaire in German. (DOCX 18 kb) [file 13063_2018_2876_MOESM1_ESM.docx]

**Additional file 1: Quality of Life Questionnaire in German:**

Die folgenden Fragen beziehen sich ausschließlich auf Ihre Augenerkrankung (endokrine Orbitopathie).
Bitte beziehen Sie sich in der Beantwortung dieser Fragen ausschliesslich auf die vergangene Woche.

In welchem Ausmaß waren während der letzten Woche die unten beschriebenen Tätigkeiten wegen der endokrinen Orbitopathie eingeschränkt?

Bitte kreuzen sie jeweils das entsprechende Kästchen an. Die Kästchen entsprechen den unten aufgeführten Antwortmöglichkeiten. Bitte geben Sie pro Frage nur eine Antwort.

|  | Ja, sehr eingeschränkt | Ja, ein bisschen eingeschränkt | Nein, überhaupt nicht eingeschränkt | | |
| --- | --- | --- | --- | --- | --- |
| Fahrradfahren Ich kann nicht Fahrradfahren |  |  |  | | |
| Autofahren Ich habe keinen Führerschein |  |  |  | | |
| Bewegung innerhalb der häuslichen Umgebung |  |  |  | | |
| Bewegung außerhalb der häuslichen Umgebung |  |  |  | | |
| Lesen |  |  |  | | |
| Fernsehen |  |  |  | | |
| Hobbies/Freizeitbeschäftigungen (welche ?..................) |  |  |  | | |
|  | Ja, sehr | Ja, ein bisschen | Nein, überhaupt nicht | | |
| Konnten Sie im Lauf der vergangenen Woche wegen der endokrinen Orbitopathie etwas nicht machen, das Sie gern getan hätten? |  |  |  | | |
| Die folgenden Fragen beziehen sich auf die endokrine Orbitopathie im Allgemeinen | | | | | |
|  | Ja, sehr | Ja, ein wenig | Nein, gar nicht | | |
| Haben Sie den Eindruck, dass sich Ihr Aussehen durch die endokrine Orbitopathie verändert hat? |  |  |  | | |
| Haben Sie den Eindruck, auf der Straße wegen Ihres durch die endokrine Orbitopathie veränderten Aussehens angestarrt zu werden? |  |  |  | | |
| Haben Sie den Eindruck, dass andere Menschen auf Sie wegen Ihrer endokrinen Orbitopathie unangenehm reagieren? |  |  |  | | |
| Glauben Sie, daß die endokrine Orbitopathie Ihr Selbstvertrauen beeinflusst? |  |  |  | | |
| Haben Sie das Gefühl, durch die endokrine Orbitopathie sozial isoliert zu |  |  |  | | |
| sein? | | |  |  |  |
| Glauben Sie, daß die endokrine Orbitopathie Ihre Fähigkeit, neue Freunde kennenzulernen, beeinflußt? | | |  |  |  |
| Haben Sie den Eindruck, daß Sie wegen der endokrinen Orbitopathie weniger häufig auf Fotografien abgebildet sind als früher? | | |  |  |  |
| Versuchen Sie, Veränderungen in Ihrem Aussehen, die durch die endokrine Orbitopathie verursacht wurden, zu verbergen? | | |  |  |  |
