# Supplementary material for: Total thyroidectomy (Tx) versus thionamides (antithyroid drugs) in patients with moderate-to-severe Graves’ ophthalmopathy – a 1-year follow-up: study protocol for a randomized controlled trial
Source: Trials. 2018 Sep 15;19:495. doi: 10.1186/s13063-018-2876-0 (PMC6139165; doi:10.1186/s13063-018-2876-0)
Supplement: Supplementary file 2 — Case report form. (DOCX 72 kb) [file 13063_2018_2876_MOESM2_ESM.docx]

# **Case Report Form**

*1. Patient data*

| Patient No: | | | | | | | | | | |
| --- | --- | --- | --- | --- | --- | --- | --- | --- | --- | --- |
| Surname: | | | | | | | | | | |
| Name: | | | | | | | | | | |
| Date of birth: | | | | | | | | | | |
| Height: | | | | | Weight: | | | | | |
| Sex: | | | | | | | | | | |
| male | | | | | | female | | | | |
| *Medical history incl. co-morbidities: | | | | | | | | | | |
| *Family history for autoimmune thyroid disease | | | No: | | | | | Yes: | | |
| Blood pressure: | | | | | | Heart rate: | | | | |
| **Pregnancy test: | | | Negative | | | | | Positive | | |
| Allergies: | | | Yes | | | | | No | | |
| Investigation: | | | | | | | | | | |
| Initial screening | 4 weeks | | | 3 mo | | | 6 mo | | | 12 mo |
| Date of investigation: | | | | | | | | | | |
| *Onset of thyroid symptoms: | | | | | | | | | | |
| *Months since diagnosis: | | | | | | | | | | |
| ***Smoking: No | | Ex-smoker | | | Current smoker  Total cigs per day: | | | | Total pack-years: | |

*2. Results of investigation*

*2.1 Blood parameters*

| **Parameter** | **Value** | **Not performed** |
| --- | --- | --- |
| TSH |  |  |
| fT3 |  |  |
| fT4 |  |  |
| TPO-Ab |  |  |
| TRAb |  |  |
| Tg |  |  |
| Calcitonin |  |  |
| PTH |  |  |
| Ca++ |  |  |
| 25-OH Vitamin D |  |  |
| 1,25 (OH)2 Vitamin D |  |  |

*3. Current Medication Day 1 ☐ Day 2 ☐ Day 3 ☐ Day 4 ☐ Day 5☐ Day 6 ☐*

*Follow-up 1 ☐ Follow-up 2 ☐ Follow-up 3 ☐, Follow-up 4 ☐ Other (date)_______ ☐*

|  | | dose/day |
| --- | --- | --- |
| Methimazole | |  |
| PTU | |  |
| T4 | |  |
| No thyroid medication: | |  |
| Lubricants: No | Yes: |  |
| Other medications: | |  |

*4. Side effects of thyroid medication or glucocorticoids:*

*4.1 Written details on side effects*

|  |
| --- |

*5. Orbitopathy*

| *Onset: | | | | | | | |
| --- | --- | --- | --- | --- | --- | --- | --- |
| *History of GO incl. symptoms: | | | | | | | |
| Current symptoms: | | | | | | | |
| Orbital pain | Gave evoked pain | | Diplopia | Blurred vision | | Grittiness/watery/photophobia | |
| Previous and current treatments: | | | | | | | |
| Systemic steroids: | | Never | | | Current IV steroids  Current oral steroids | | Previous steroids  Months since stopping |
| Orbital radiation: | | No | | | Yes  Completed: | |  |
| Surgery for GO: | | No | | | Yes  Date: | |  |
| Selenium: | | No | | | Yes | |  |
| Other treatments: | | No | | | Yes | |  |

*5.1 Examination of eyes*

|  | | | | | Right | | | | | | | | Left | | | | | | | |
| --- | --- | --- | --- | --- | --- | --- | --- | --- | --- | --- | --- | --- | --- | --- | --- | --- | --- | --- | --- | --- |
| (Corrected) visual acuity  Refraction | | | | |  | | | | | | | |  | | | | | | | |
|  |  |  |  |  |  | | | | | | | |  | | | | | | | |
| Relative Afferent Pupil Defect | | | | | No | | Yes | | | | | | No | | Yes | | | | | |
| Soft tissues | Active lid swelling | | | Upper | Unsure Nil or equivocal  moderate severe | | | | | | | | Unsure Nil or equivocal  moderate severe | | | | | | | |
|  |  |  |  | Lower | Unsure Nil or equivocal  moderate severe | | | | | | | | Unsure Nil or equivocal  moderate severe | | | | | | | |
|  | Lid erythema | | | Upper | No | | | Yes | | | | | No | | | Yes | | | | |
|  |  |  |  | Lower | No | | | Yes | | | | | No | | | Yes | | | | |
|  | Conjunctival redness | | | | No | | Yes | | | | | | No | | Yes | | | | | |
| Lid positions | Palpebral aperture | | | |  | | | | | | | |  | | | | | | | |
|  | Upper lid retraction | | | |  | | | | | | | |  | | | | | | | |
|  | Lower lid retraction | | | |  | | | | | | | |  | | | | | | | |
|  | Levator function | | | |  | | | | | | | |  | | | | | | | |
|  | Bells’ phenomenon | | | | No | | Yes | | | | | | No | | Yes | | | | | |
|  | Lagophthalmos | | | | No | | Yes | | | | | | No | | Yes | | | | | |
|  | Is cornea exposed | | | | No | Slight | | | | | | Yes | No | Slight | | | | | | Yes |
| Proptosis | Intercanthal distance | | | |  | | | | | | | |  | | | | | | | |
| Motility | | | | |  | | | | | | | |  | | | | | | | |
| Slit lamp | | | Chemosis | | No | | | | | | | | Yes | | | | | | | |
|  |  |  | Carunle or plical inflamm. | | No | | | | | | | | Yes | | | | | | | |
| Cornea | | |  | | Normal erosions | Ulcer | | | | | | Perforation | Normal erosions | Ulcer | | | | | | Perforation |
| Intraocular pressure in primary gaze | | | | |  | | | | | | | |  | | | | | | | |
| Fundoscopy | | Disc | | | Normal | Atrophy | | | Swollen | | | other | Normal | Atrophy | | | Swollen | | | other |
|  | | Choroidal folds | | | No | | | | | | Yes | | No | | | | | | Yes | |
| Any evidence of optic neuropathy | | | | | No | | | | | Yes | | | No | | | | | Yes | | |

*Only required at initial screening

** Only required at initial screening and before admittance of any glucocorticoid therapy

*** Only required at initial screening and at the last control appointment

*5.2 Clinical activity score:*

| Active lid swelling | ☐ |
| --- | --- |
| Eyelid erythema | ☐ |
| Definite conjunctival redness | ☐ |
| Chemosis | ☐ |
| Caruncle or plical inflammation | ☐ |
| Gaze evoked orbital pain | ☐ |
| Spontaneous orbital pain | ☐ |

**Total CAS /7**

*5.3* ***Active GO:*** No: ☐ Yes: ☐

*6. Further comments (optional)*

|  |
| --- |

*7. Study continuation/discontinuation:*

| Continuation of study therapy |  |
| --- | --- |
| Discontinuation of study therapy |  |
| **Date of discontinuation:** | |
| Reasons for discontinuation | |
| Patient’s withdrawal due to side effects |  |
| Patient’s withdrawal due to other reason | Reason: |
| Medical indication due to side effects |  |
| Medical indication due to other reason | Reason: |
